# Association between serum levels of Klotho and inflammatory cytokines in cardiovascular disease: a case-control study

**DOI:** 10.18632/aging.102734

**Published:** 2020-01-27

**Authors:** Ernesto Martín-Núñez, Javier Donate-Correa, Carla Ferri, Ángel López-Castillo, Alejandro Delgado-Molinos, Carolina Hernández-Carballo, Nayra Pérez-Delgado, Sergio Rodríguez-Ramos, Purificación Cerro-López, Víctor G. Tagua, Carmen Mora-Fernández, Juan F. Navarro-González

**Affiliations:** 1 Research Unit, University Hospital Nuestra Señora de Candelaria, Santa Cruz de Tenerife, Spain; 2 Doctoral and Graduate School, University of La Laguna, San Cristóbal de La Laguna, Spain; 3 Vascular Surgery Service, University Hospital Nuestra Señora de Candelaria, Santa Cruz de Tenerife, Spain; 4 Clinical Analysis Service, University Hospital Nuestra Señora de Candelaria, Santa Cruz de Tenerife, Spain; 5 Transplant Coordination, University Hospital Nuestra Señora de Candelaria, Santa Cruz de Tenerife, Spain; 6 Nephrology Service, University Hospital Nuestra Señora de Candelaria, Santa Cruz de Tenerife, Spain; 7 Institute of Biomedical Technologies, University of La Laguna, San Cristóbal de La Laguna, Spain

**Keywords:** Klotho, cardiovascular disease, inflammation, TNFα, IL10

## Abstract

Decrease in soluble anti-aging Klotho protein levels is associated to cardiovascular disease (CVD). Diverse studies have shown a bidirectional relationship between Klotho and inflammation, a risk factor for the development of CVD. In this work we aimed to evaluate the association between Klotho and inflammatory cytokines levels in the context of human CVD.

The study included 110 patients with established CVD and preserved renal function, and a control group of 22 individuals without previous history of cardiovascular events. Serum Klotho and IL10 levels were significantly lower in the CVD group. Inflammatory status, marked by the TNFα/IL10 ratio and the C-reactive protein (CRP) levels, was significantly increased in the group of patients with established CVD. Soluble Klotho levels were directly correlated with eGFR (r=0.217) and IL10 (r=0.209) and inversely correlated with age (r=-0.261), CRP (r=-0.203), and TNFα/IL10 (r=-0.219). This association with TNFα/IL10 remained significant in age-matched subgroups. Multiple logistic regression analysis showed that age, smoking and the neutrophil-to-lymphocyte ratio (NLR) constituted risk factors for the presence of CVD, while Klotho was a protective factor.

In conclusion, in patients with established CVD, the reduction in soluble Klotho is associated with a pro-inflammatory status marked by lower IL10 concentrations and higher TNFα/IL10 ratio and CRP levels.

## INTRODUCTION

*Klotho (KL)* is a longevity-related gene discovered in 1997 [1]. Defects in *KL* expression leads to a syndrome resembling human aging that includes endothelial dysfunction, vascular calcification and progressive atherosclerosis [1]. *KL* gene encodes a single-pass transmembrane glycoprotein type 1, mainly expressed in renal tubular cells, and a soluble form derived from the action of membrane proteases that cleave the extracellular domain of the membrane-bound protein [2, 3].

Aging is associated with a reduction in the renal expression and serum concentrations of Klotho, which is also observed in diseases characterized by premature vascular aging, such as renal failure, hypertension, and diabetes mellitus [4–6]. Moreover, several clinical studies have observed that lower systemic levels of the soluble form of Klotho are associated with markers of vascular dysfunction [7–10] as well as with the prevalence and severity of cardiovascular disease (CVD) [11–13]. These associations are particularly relevant in renal patients since the largest proportion of systemic Klotho is generated by the kidneys and its levels are reduced along with the renal function decline [14].

Chronic inflammation is an important factor in the development of CVD. Increased serum levels of different acute phase proteins and inflammatory cytokines, as well as infiltration of the vascular tissue by immune cells, are characteristics of a low-grade inflammation scenario which may act as substrate for the development and progression of atherogenic damage. Different inflammatory cytokines, including tumor necrosis factor alpha (TNFα) and interleukin (IL) 10, are involved in the onset and development of the vascular lesion [15–16]. Interestingly, inflammatory cytokines can down-regulate Klotho levels [17–18] whereas, in the opposite direction, Klotho is able to modulate inflammation by inhibiting central signaling pathways and the expression of inflammatory related molecules [19–21]. This raises the intriguing question about the potential interrelationships between Klotho and systemic inflammation in the context of human CVD, an aspect that has not been previously addressed. Therefore, the aim of this work is to analyze, in a group of patients with established CVD and preserved renal function, the serum levels of soluble Klotho and its association with the concentration of inflammatory cytokines related to atherosclerotic vascular damage.

## RESULTS

From the 181 potential subjects who were initially considered in the CVD group, 71 were excluded according to exclusion criteria and 110 patients were finally included (Supplementary Figure 1). The demographic, clinical and biochemical data of the entire study population, included the control group, are presented in Table 1. The clinical diagnosis consisted of peripheral artery disease (PAD) (66.4%), transient ischemic attack (TIA) (39.1%), and abdominal aortic aneurysm (AAA) (18.2%). The CVD group was significantly older (*P*<0.01), with a higher prevalence in hypertension (HT) (*P*<0.05) but not in diabetes mellitus (DM). This group presented a lower estimated glomerular filtration rate (eGFR) (*P*<0.05), as well as lower serum concentrations of calcium (*P*<0.001). The use of antiaggregants (*P*<0.0001), angiotensin-converting enzyme inhibitors or angiotensin receptor antagonist (*P*<0.05) and statins (*P*<0.0001) was significantly higher in the CVD-group.

**Table 1:** Clinical characteristics and biochemical assessments of the patients included in the study.

**Variable**	**CVD (n=110)**	**Non-CVD (n=22)**	***P*-value**
***Characteristics***			
Age (years)	66.7±9.5	59.1±12.2	<0.01
Sex (M/F)	88 / 22	13 / 9	NS
Smoker (%)	77.3	63.6	NS
Alcoholism (%)	47.3	36.4	NS
BMI (kg/m^2^)	28.1±4.3	28.7±3.1	NS
***Comorbidities***			
HT (%)	79.1	54.6	<0.05
DM (%)	48.2	36.4	NS
**CVD**			
PAD (%)	66.4	0	<0.0001
TIA (%)	39.1	0	<0.0001
AAA (%)	18.2	0	<0.05
***Pharmacological treatment***			
Antiaggregants (%)	86.4	18.2	<0.0001
Beta-blockers (%)	28.2	13.6	NS
ACEI/ARA2 (%)	51.8	22.7	<0.05
CCB (%)	22.7	13.6	NS
Statins (%)	84.6	4.6	<0.0001
***Laboratory data***			
eGFR (mL/min/1.73m^2^)	89.8±13	98.0±15.6	<0.05
Creatinine (mg/dL)	0.83±0.2	0.76±0.1	0.08
Albumin (g/dL)	3.9±0.6	4.1±0.5	0.13
Calcium (mg/dL)	9.1±0.5	9.5±0.5	<0.001
Phosphorus (mg/dL)	3.5±0.5	3.5±0.8	NS
Uric acid (mg/dL)	5.8±1.4	6.1±1.4	NS
Glucose (mg/dL)	111.7±29.1	116.8±33.8	NS
Total cholesterol (mg/dL)	165.7±43.3	186.0±33.2	0.06
HDL (mg/dL)	43.8±11.0	48.2±16.0	NS
NLR	3.7±2.1	2.9±1.4	0.19
CRP (mg/dL)	5.3±4.8	1.5±0.7	<0.0001
Klotho (pg/mL)	487.8 (357-645.7)	954.1 (605.1-1365)	<0.001
TNFα (pg/mL)	1.11 (0.81-1.50)	1.46 (0.78-2.0)	NS
IL10 (pg/mL)	3.91 (0.60-7.25)	10.30 (3.94-25.14)	<0.01
TNFα/IL10	0.39 (0.15-1.82)	0.16 (0.06-0.45)	<0.01

BMI, body mass index; HT, hypertension; DM, diabetes mellitus; PAD, peripheral artery disease; TIA, transient ischemic attack; AAA, abdominal aortic aneurysm; ACEI/ARA2, angiotensin converting enzyme inhibitor/angiotensin receptor antagonist 2; CCB, calcium channels blockers; eGFR, estimated glomerular filtration rate; HDL, high-density lipoprotein; NLR, neutrophil-to-lymphocyte ratio; CRP, C-reactive protein; TNFα, tumor necrosis factor alpha; IL10, interleukin 10; NS, not significant.

### Serum levels of soluble Klotho and inflammatory cytokines in CVD.

**Figure 1:**
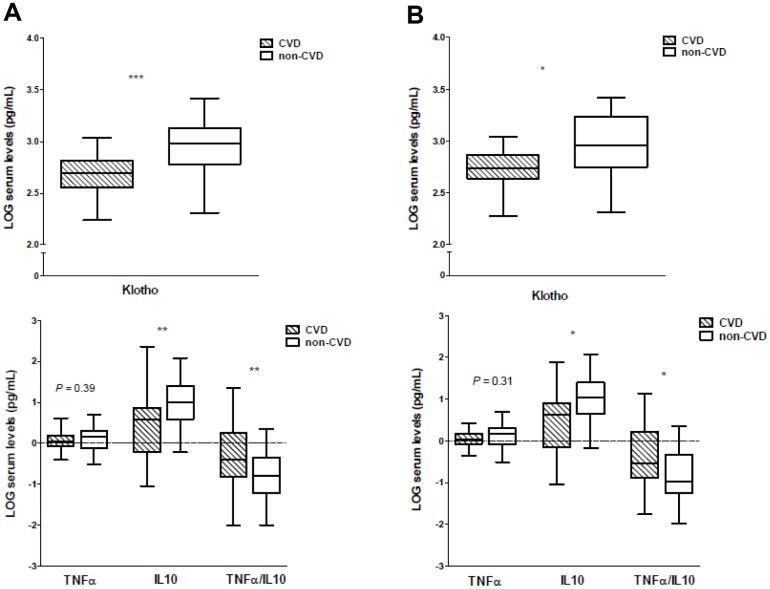
**Soluble Klotho and inflammatory cytokines serum levels in CVD and non-CVD groups.** (**A**) Full-study groups, (**B)** age-matched subgroups. *P<0.05, ** *P*<0.01, ***P<0.001.

Soluble Klotho concentrations were significantly lower in patients with established CVD (*P*<0.001) (Table 1 and Figure 1A), without differences according to each specific form of clinical atherosclerotic vascular disease (*P*=0.47). Regarding the inflammatory cytokines, there were no significant differences concerning serum TNFα levels (*P*=0.39). However, the serum concentrations of IL10 were significantly higher in the control group (*P*<0.01). In order to better evaluate the inflammatory status of the subjects, understood as the balance between pro- and anti-inflammatory forces, we calculated the ratios between the serum levels of TNFα, a pro-inflammatory factor, to IL10 concentrations, a cytokine with anti-inflammatory effects. A significantly higher TNFα/IL10 ratio was observed in the CVD group compared with controls (*P*<0.01) (Figure 1A). No differences in serum levels of these inflammatory cytokines or in the TNFα/IL10 ratio were observed according to the forms of clinical vascular disease. Inflammatory status was also evaluated by the serum concentration of C-reactive protein (CRP) and the neutrophil-to-lymphocyte ratio (NLR). Both parameters were higher in the CVD group, although only the differences for CRP reached statistical significance (*P*<0.0001), while the mean NLR showed a trend (*P*=0.19) (Table 1). Most of the patients were receiving antiaggregants, renin-angiotensin system blockers or statins. When evaluating the serum levels of Klotho or the inflammatory cytokines studied between these subjects who received treatment and those who did not, we found no significant differences.

The characteristics of patients in the CVD group stratified by tertiles of soluble Klotho are shown in Table 2. Subjects within the highest tertile of soluble Klotho showed lower serum concentrations of TNFα (*P*<0.05) and tended to present decreased levels of TNFα/IL10 ratio (*P*=0.09).

**Table 2:** Clinical characteristics and biochemical assessments of patients with established CVD stratified by tertiles of serum Klotho levels.

**Variable**	**Tertile 1(<391.1 pg/mL) (n=37)**	**Tertile 2(391.1-591.8 pg/mL) (n=37)**	**Tertile 3(>591.8 pg/mL) (n=36)**	***P*-value**
***Characteristics***				
Age (years)	67.7±9.3	68.3±8.6	64.2±8.6	NS
Sex (M/F)	31 / 6	27 / 10	30 / 6	NS
Smoking (%)	81.1	75.7	73	NS
Alcoholism (%)	51.4	37.8	51.4	NS
BMI (kg/m^2^)	29.0±4.1	27.8±4.1	27.4±4.6	NS
***Co-morbidities***				
HT (%)	81.1	81.1	75.0	NS
DM (%)	54.1	54.0	36.1	NS
***Pharmacological treatment***				
Antiaggregants (%)	83.8	86.5	88.9	NS
Beta-blockers (%)	32.4	27.1	25.0	NS
ACEI/ARA2 (%)	51.4	59.5	44.4	NS
CCB (%)	16.2	32.4	19.4	NS
Statins (%)	89.2	89.2	75.0	NS
***Laboratory data***				
eGFR (mL/min/1.73m^2^)	88.7±11.2	87.4±13.4	93.0±13.4	0.13
Creatinine (mg/dL)	0.84±0.2	0.84±0.2	0.81±0.2	NS
Albumin (g/dL)	4.0±0.6	3.8±0.6	3.9±0.6	NS
Calcium (mg/dL)	9.1±0.5	9.2±0.5	9.2±0.4	NS
Phosphorus (mg/dL)	3.5±0.5	3.5±0.4	3.6±0.5	NS
Uric acid (mg/dL)	5.87±1.6	6.2±1.3	5.4±1.3	NS
Glucose (mg/dL)	110.4±26.1	113.5±32.5	111.2±28.9	NS
Total cholesterol (mg/dL)	157.6±48.1	169.4±40.9	171.8±54.9	NS
HDL (mg/dL)	42.3±12.8	43.9±10.4	44.1±11.8	NS
NLR	3.7±2.1	3.8±2.2	3.6±1.9	NS
CRP (mg/dL)	4.1±2.9	3.5±2.8	3.2±2.6	NS
TNFα (pg/mL)	1.13 (0.88-1.65)	1.02 (0.82-1.35)	1.02 (0.69-1.39)	<0.05
IL10 (pg/mL)	1.05 (0.51-5.81)	2.17 (0.55-4.9)	4.03 (0.58-5.19)	NS
TNFα/IL10	0.73 (0.26-1.81)	0.63 (0.19-1.77)	0.28 (0.15-1.99)	0.09

BMI, body mass index; HT, hypertension; DM, diabetes mellitus; ACEI/ARA2, angiotensin converting enzyme inhibitor/angiotensin receptor antagonist 2; CCB, calcium channels blockers; eGFR, estimated glomerular filtration rate; HDL, high-density lipoprotein; NLR, neutrophil-to-lymphocyte ratio; CRP, C-reactive protein; TNFα, tumor necrosis factor alpha; IL10, interleukin 10; NS, not significant.

To avoid the confounding effect of age on serum Klotho levels, we performed a subanalysis in our cohort by age-matching the study groups (Supplementary Figure 1). The clinical and laboratory data of these subgroups are presented in Supplementary Table 1. Again, serum levels of soluble Klotho were significantly lower in the group of participants with established CVD (*P*<0.05) (Figure 1B). There were no differences in serum Klotho concentration among patients according to the presence of PAD or TIA. The serum levels of TNFα did not show significant differences between both groups (*P*=0.31), whereas in the non-CVD group the serum IL10 concentrations were significantly higher (*P*<0.05) and the values of the TNFα/IL10 ratio were significantly lower (*P*<0.05) (Figure 1B). No differences in serum levels of these inflammatory cytokines were observed among the patients according to the presence of PAD or TIA. Serum levels of CRP and the NLR showed higher values in the CVD group, differences that almost reached statistical significance (*P*=0.08 and *P*=0.10, respectively).

### Associations between soluble Klotho levels and other factors

Correlation analysis for soluble Klotho levels and other factors are shown in Table 3A. The analysis revealed that soluble Klotho levels were significantly associated with age, eGFR, serum levels of CRP and IL10, and with the TNFα/IL10 ratio. When the analysis was performed in the age-matched subgroup, the associations between soluble Klotho with IL10, as well as with the TNFα/IL10 ratio remained significant. Moreover, a multiple regression analysis using soluble Klotho levels as the dependent variable and age, sex, smoking, HT, DM, and serum levels of total cholesterol, phosphorus, uric acid, NLR and TNFα/IL10 ratio as covariates (Table 3B) showed that age (β=-0.234, *P*<0.05) and TNFα/IL10 ratio (β=-0.243, *P*<0.01) were significantly associated with soluble Klotho concentration in the full-study cohort (adjusted R^2^: 0.068, *P*<0.05), while only the association with the TNFα/IL10 ratio remained significant in the age-matched subgroup (adjusted R^2^: 0.232, *P*<0.05).

**Table 3:** Association between soluble Klotho levels and other factors.

**A) Univariate correlation analysis**	**Full-study group (n=132)**		Age-matched subgroup* (n=60)	
**Variable**	***r***	***P*-value**	***r***	***P*-value**
**Age**	-0.261	<0.01	0.047	NS
**BMI**	-0.134	0.13	-0.255	0.06
**eGFR**	0.217	<0.05	0.250	0.07
**Creatinine**	-0.138	0.12	-0.160	NS
**Albumin**	-0.026	NS	-0.087	NS
**Calcium**	0.153	0.08	0.172	NS
**Phosphorus**	0.110	NS	0.130	NS
**Uric acid**	0.041	NS	0.219	0.12
**Glucose**	0.075	NS	0.140	NS
**Total cholesterol**	0.163	0.07	0.248	0.08
**HDL**	0.039	NS	0.162	NS
**NLR**	-0.015	NS	0.172	NS
**CRP**	-0.203	<0.05	-0.235	0.13
**TNFα**	-0.035	NS	0.074	NS
**IL10**	0.209	<0.05	0.278	<0.05
**TNFα/IL10**	-0.219	<0.05	-0.256	<0.05

**B) Multiple regression analysis**	**Full-study group** **(n=132)**					Age-matched subgroup*(n=60)				
**Variable**	**Adj-R^2^**	**β**	**SE**	**t**	***P*-value**	**Adj-R^2^**	**β**	**SE**	**t**	***P*-value**
***Adjusted R^2^***	0.068				<0.05	0.232				<0.05
**Age**		-0.234	0.002	-2.327	<0.05		0.065	0.007	0.346	NS
**Sex**		-0.006	0.048	-0.067	NS		-0.124	0.073	-0.636	NS
**Total cholesterol**		0.067	0.000	0.714	NS		0.345	0.001	1.937	0.07
**NLR**		0.058	0.009	0.653	NS		0.262	0.035	1.414	0.17
**TNFα/IL10**		-0.243	0.026	-2.621	<0.01		-0.411	0.059	-2.355	<0.05
**Smoker**		-0.105	0.047	-1.124	NS					
**HT**		-0.10	0.049	-0.105	NS					
**DM**		-0.10	0.041	-0.102	NS					
**Phosphorus**		0.046	0.040	0.518	NS					
**Uric acid**		0.19	0.014	0.209	NS					

BMI, body mass index; eGFR, estimated glomerular filtration rate; HDL, high-density lipoprotein; NLR, neutrophil-to-lymphocyte ratio; CRP, c-reactive protein; TNFα, tumor necrosis factor alpha; IL10, interleukin 10; HT, hypertension; DM, diabetes mellitus; NS, not significant. *_ Age-matched subgroup (2 CVD:1 non-CVD).

### Logistic regression analysis of predictors of CVD

The results of the multivariate logistic regression analysis, using the presence/absence of CVD as the dependent variable, are presented in Table 4. Traditional risk factors for CVD (age, sex, smoking, HT, DM, serum cholesterol and eGFR) were entered as covariates, with additional models in which NLR, serum CRP and TNFα/IL10 levels (model 2), and soluble Klotho concentrations (model 3) were added as covariates. In model 1, only age and smoking were independent risk factors for CVD. In model 2, age, smoking and NLR were risk factors significantly associated with CVD. Finally, in model 3, in addition to the previous variables, soluble Klotho serum level was an independent protective factor for the presence of CVD.

**Table 4:** Multivariate logistic regression analysis for the presence of CVD.

**Independent variable**	**OR (95% CI)**	***P*-value**
***Model 1***		
Age	1.086 (1.011 to 1.166)	<0.05
Sex	0.262 (0.075 to 1.914)	0.19
Smoker	3.216 (1.810 to 12.773)	<0.05
HT	0.814 (0.203 to 3.261)	NS
DM	0.773 (0.230 to 2.603)	NS
Serum cholesterol	0.988 (0.975 to 1.001)	0.12
eGFR	0.961 (0.916 to 1.007)	0.09
***Model 2***		
Age	1.203 (1.044 to 1.386)	<0.05
Sex	5.313 (0.545 to 51.778)	0.15
Smoker	55.448 (3.183 to 965.866)	<0.01
HT	0.511 (0.060 to 4.337)	NS
DM	0.622 (0.088 to 4.415)	NS
Serum cholesterol	0.976 (0.954 to 1.998)	0.09
eGFR	0.986 (0.926 to 1.051)	NS
CRP	1.522 (0.972 to 2.383)	0.07
NLR	1.804 (1.028 to 3.166)	<0.05
TNFα/IL10	14.726 (0.945 to 229.372)	0.06
***Model 3***		
Age	1.336 (1.059 to 1.685)	<0.05
Sex	7.132 (0.302 to 168.594)	NS
Smoker	55.658 (1.543 to 2007.350)	<0.05
HT	0.951 (0.047 to 19.267)	NS
DM	3.721 (0.137 to 100.783)	NS
Serum cholesterol	0.968 (0.938 to 1.019)	0.06
eGFR	1.058 (0.965 to 1.159)	NS
CRP	1.178 (0.723 to 1.919)	0.13
NLR	5.524 (1.096 to 31.709)	<0.05
TNFα/IL10	27.552 (0.608 to 1246.780)	0.08
Klotho	0.995 (0.991 to 0.998)	<0.01

HT, hypertension; DM, diabetes mellitus; eGFR, estimated glomerular filtration rate; CRP, c-reactive protein; NLR, neutrophil-to-lymphocyte ratio; TNFα, tumor necrosis factor- α; IL10, interleukin 10; NS, not significant.

## DISCUSSION

In this study, we observed that the decrease in soluble Klotho levels that occurs in CVD (defined as PAD, TIA or AAA) is associated with the systemic inflammatory environment that accompanies this disorder. We determined the serum levels of TNFα and IL10, inflammatory cytokines involved in the onset and development of vascular disease, in addition to other inflammatory parameters such as CRP or NLR. In particular, we observed significantly higher values of CRP and TNFα/IL10 ratio in patients with established CVD, which also presented significantly lower levels of soluble Klotho. The difference between groups for the TNFα/IL10 ratio was due to a reduction in the IL10 serum levels rather than an increase in the TNFα serum concentration. Furthermore, we observed that soluble Klotho levels are directly associated to IL10, while there is an inverse correlation with CRP and TNFα/IL10 ratio. Interestingly, when we matched both groups by age, a factor associated with soluble Klotho levels [22], the correlations between Klotho and the levels of IL10 and the TNFα/IL10 ratio still remained significant. The patients with higher values of serum Klotho showed a trend to have decreased values of the TNFα/IL10 ratio. Moreover, we observed that while age and NLR were independent risk factors for the presence of CVD, higher serum Klotho levels were associated with a lower risk.

The results of this work agree with previous clinical studies that have observed a reduction in soluble Klotho levels in patients with CVD. In 2011, Semba et al. showed in a cohort of 1023 community-dwelling adults that higher plasma Klotho levels were independently associated with a lower likelihood of having CVD (understood as coronary artery disease (CAD), heart failure, stroke or PAD and, similarly to our study, they observed an inverse association between circulating Klotho and CRP levels (*r*=-0.10, *P*<0.001) [11]. Besides, we previously reported that patients with significant CAD present lower soluble Klotho levels and, also, that soluble Klotho concentration is independently and negatively associated with the severity of the coronary stenosis [12]. Interestingly, several studies have pointed out a relationship between the decrease in serum soluble Klotho and worse outcomes in vascular functional tests, like reduced capacity of flow-mediated dilation of the brachial artery, or higher values of epicardial fat thickness and carotid artery intima-media thickness (cIMT) in healthy adults [8], or increased ankle-brachial pulse wave velocity, left ventricular mass index, cIMT and carotid atherosclerotic plaque quantity in chronic kidney disease population [5–7]. Furthermore, Pan et al. recently showed, in a prospective study of 252 patients and a 7-year follow-up, that lower baseline levels of circulating Klotho were associated with an increased risk of developing CAD or cerebrovascular accident (CVA) in the long term in diabetic population [13]. Despite the multiple evidences that point to CVD as a state of soluble Klotho deficiency, a definite cause for this decrease is not yet known. A plausible explanation is a decrease in the renal production either by a repression of the *KL* gene or by a reduction of the expression/activity of the metalloproteases responsible for generating the soluble form. Unfortunately, there are no studies that address these issues. A factor closely related to Klotho expression is renal function, so it is known that in CKD it occurs a reduction in renal and systemic Klotho levels (14). In fact, there are several works that relate a lower production of soluble Klotho in renal patients with a worse vascular function [5–7]. However, in our study we show how this decrease in soluble Klotho during CVD also occurs in individuals with a preserved renal function (all of them with eGFR>60 mL/min/1.73m^2^; mean eGFR 89.8 ±13mL/min/1.73m^2^), so there must be others factors beyond the renal status that affect serum Klotho levels.

Inflammation, that usually accompanies CVD, may has some role in the modulation of systemic Klotho. Although all previous studies have provided new insights into the role of soluble Klotho in the development of CVD, none of them have inquired in the potential interactions between Klotho and inflammatory cytokines. We have addressed this question for the first time in this study. The expression of Klotho can be negatively modulated by inflammatory cytokines, such as TNFα or tumor necrosis factor-like weak inducer of apoptosis (TWEAK) [17, 18]. Though in our study we did not observe differences in serum levels of TNFα or an association between soluble Klotho and this pro-inflammatory cytokine, we found an inverse correlation with CRP, another pro-inflammatory marker. However, it is interesting to highlight the potential anti-inflammatory effects of Klotho [19–21]. In our results, we observed a direct relationship between serum Klotho levels and IL10 concentrations, both decreased in patients with CVD. This association could suggest that, partially, the effects on vascular homeostasis in which Klotho participates could be through modulation of anti-inflammatory cytokine levels. Interestingly, we found that this relationship between Klotho and IL10 remained after eliminating the effect of age in our study groups, which is a factor influencing Klotho levels. This might suggest that the role of Klotho in CVD and its relationship with inflammation is independent of age.

Limitations in our study include that, due to logistical reasons, we did not measure levels of other molecules implicated in the inflammatory response with known roles in the framework of CVD, namely IL1, IL6 or transforming growth factor (TGF) β, and therefore we cannot have a broad view of Klotho interactions with different inflammatory mediators. Likewise, we do not have either measurement of molecules of mineral metabolism related to Klotho and with potential impact on CVD, such as fibroblast growth factor 23 or vitamin D, so we cannot rule out their interactions in the Klotho-inflammation relationship. Moreover, a considerable proportion of patients received treatment with statins, a drug with known anti-inflammatory properties, that may interfere with the outcomes of our work. Although we did not observe significant differences for the parameters studied between individuals who received the treatment and those who did not, the possibility of its influence should not be completely ruled out. A further limitation is that we employed a relatively small sample size that could make that some of our results did not reach statistical significance and, consequently, the ability to describe the association between some variables might be limited. Also, this limitation could make that the findings made in these groups not transferable to larger populations. Finally, due to the cross-sectional design of the study we can detect associations but not to infer causality.

## CONCLUSION

Soluble Klotho is decreased in CVD and its decline is associated to the pro-inflammatory background set during disease. Our results are in line with previous findings in the literature that point to soluble Klotho as a protective factor against cardiovascular disorders. Further works that deepen into the interactions between Klotho and systemic inflammatory mediators, with particular interest in IL10, are needed and may offer new strategies for the treatment of vascular pathologies.

## MATERIALS AND METHODS

### Patients

Two groups of participants were consecutively enrolled from November 2014 to September 2017 at the University Hospital Nuestra Señora de Candelaria (UHNSC – Santa Cruz de Tenerife, Spain). In the case group, a total of 181 adult patients undergoing an elective open vascular surgery procedure due to clinical atherosclerotic vascular disease were enrolled. Exclusion criteria for this group included hemodynamic instability during the surgical procedure (defined as a systolic blood pressure lower than 90 mm Hg or the need for inotropes or vasopressors); history of chronic inflammatory, immunologic, or tumoral disease; positive serology to hepatitis B, hepatitis C, or HIV; acute inflammatory or infectious intercurrent episodes in the previous month; institutionalization; chronic renal disease, defined as an eGFR lower than lower than 60 mL/min/1.73m^2^; receipt of immunotherapy or immunosuppressive treatment; and inability or unwillingness to provide informed consent. The control group was formed by 22 cadaveric organ donors without any medical antecedent or study showing the presence of CVD. From this initial study population, a subgroup was developed by randomly age-matching individuals from both groups in a 2 CVD patient:1 non-CVD subject ratio. The study protocol was approved by the Institutional Ethics Committee of the UHNSC and complied with ethical standards of the Declaration of Helsinki. Written informed consent was obtained from all participants or their families, in the case of the control group.

### Biochemical parameters

Serum samples were drawn at the time of surgery or organ retrieval, aliquoted and immediately frozen at −80ºC. Routine biochemical parameters were measured using standard methods. Concentrations of serum Klotho protein were measured by a solid phase sandwich ELISA using the human soluble α-Klotho assay kit (Immuno-Biological Laboratories, Takasaki, Japan) according to manufacturer’s instructions. The assay sensitivity was 6.15 pg/mL and the intra and interassay coefficients of variation were 2.7-3.5% and 2.9-11.4%, respectively. The serum levels of the inflammatory cytokines TNFα and IL10 were measured by commercial ELISA assay kits: Human TNFα Quantikine HS ELISA HSTA00D and Human IL10 Quantikine D100B, respectively (R&D Systems, MN, USA). The samples with serum IL10 values under the assay range were reanalysed with the high sensitivity commercial assay Human IL10 Quantikine HS HS100C (R&D Systems, MN, USA). The sensitivity, intra and interassay coefficients of variation were, respectively: 0.106 pg/mL, 3.1-8.7% and 7.2-10.4% for the TNFα ELISA kit; 3.9 pg/mL, 1.7-5.0% and 5.9-7.5% for the IL10 assay; and 0.09 pg/mL, 4.6-9.3% and 7.8-13.1% for the IL10 high sensitivity assay. CRP was measured was measured by a high sensitivity particle enhanced immunoturbidimetric fully automated assay (Roche Diagnostics GmbH, Mannheim, Germany) in a Cobas 6000 analyzer from the same manufacturer (functional sensitivity was 0.3 mg/L and the intra- and interassay precision were 1.6 and 8.4 respectively).

### Statistical analysis

Continuous variables are reported as mean±standard deviation (SD), except for Klotho and inflammatory cytokines, which are expressed as medians and interquartile ranges (IQR). Categorical data are presented as percentages. Normal distribution of data was assessed by a D’Agostino–Pearson test. Due to non-normal distribution, serum values of Klotho and inflammatory cytokines were logarithmically transformed for statistical analysis. Differences among groups were analysed by unpaired t test, Mann-Whitney test or one-way analysis of variance with Bonferroni post hoc test. Categorical variables were compared between groups using chi-squared test or Fisher exact test. Correlation analysis of serum Klotho concentrations with different clinical and serum factors was evaluated by Spearman correlation test. Forward stepwise multiple regression analysis was performed to determine the independent association between patient clinical parameters as potential predictor variables (age, sex, HT, DM, smoking, eGFR, cholesterol, body mass index (BMI), serum uric acid, phosphorus and, NLR, CRP and TNFα/IL10) and serum Klotho concentrations as the dependent variable. A multiple logistic regression was performed to assess independent predictors of the presence of CVD. For this purpose, we adopted three models: in model 1, we introduced conventional risk factors (age, sex, smoking, HT, DM, serum cholesterol and eGFR); in model 2, we additionally included the NLR and the ratio of serum TNFα/IL10 levels; finally, in model 3, we adjusted the analysis for the soluble Klotho concentrations. Values of *P*<0.05 were considered significant. Statistical analyses were performed using IBM SPSS Statistics V.19 (IBM Corporation, NY, USA) and GraphPad Prism 6.01 software (GraphPad Software, CA, USA).

## Supplementary Material

Supplementary Figure 1

Supplementary Table 1
