# Supplementary figures and images for: Association between serum levels of Klotho and inflammatory cytokines in cardiovascular disease: a case-control study
Source: Aging (Albany NY). 2020 Jan 27;12(2):1952–64. doi: 10.18632/aging.102734 (PMC7053623; doi:10.18632/aging.102734)

SUPPLEMENTARY FIGURE

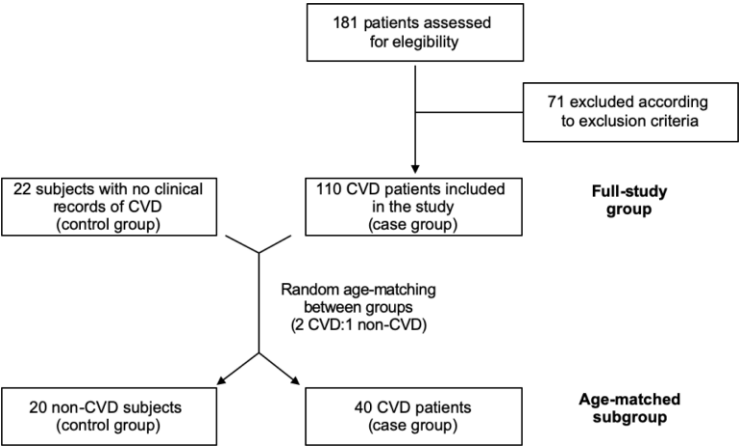

Supplementary Figure 1. Study design.

Supplement: Supplementary Figure 1 [file aging-12-102734-s001..pdf]
