# Supplementary material for: Association between serum levels of Klotho and inflammatory cytokines in cardiovascular disease: a case-control study
Source: Aging (Albany NY). 2020 Jan 27;12(2):1952–64. doi: 10.18632/aging.102734 (PMC7053623; doi:10.18632/aging.102734)
Supplement: Supplementary Table 1 [file aging-12-102734-s002..pdf]

SUPPLEMENTARY TABLE

Supplemental Table 1. Clinical and laboratory data in the age-matched subgroups.

Variable	CVD (n=40)	Non-CVD (n=20)	P-value
Age (years)	63.8±7.7	63.7±8.2	NS
Sex (M/F)	31 / 9	10 / 7	NS
Smoker (%)	78.4	58.8	NS
Alcoholism (%)	56.8	29.4	0.08
BMI (kg/m^2)	28.0±4.0	28.7±3.2	NS
Co-morbilities			
HT (%)	78.4	70.6	NS
DM (%)	48.6	35.3	NS
CVD			
PAD (%)	56.8	0	<0.0001
TIA (%)	40.5	0	<0.01
AAA (%)	10.8	0	NS
Pharmacological treatment			
Antiaggregants (%)	91.9	23.5	<0.0001
Beta-blockers (%)	24.3	11.8	NS
ACEI/ARA2 (%)	48.6	29.4	NS
CCB (%)	21.6	17.6	NS
Statins (%)	89.2	5.9	<0.0001
Laboratory data			
eGFR (mL/min/1.73m^2)	93.4±13.4	99.5±15.7	0.16
Creatinine (mg/dL)	0.80±0.2	0.77±0.1	NS
Albumin (g/dL)	3.9±0.6	4.1±0.6	NS
Calcium (mg/dL)	9.2±0.3	9.5±0.5	<0.05
Phosphorus (mg/dL)	3.6±0.5	3.5±0.9	NS
Uric acid (mg/dL)	5.9±1.3	5.8±1.3	NS
Glucose (mg/dL)	124.7±35.7	114.2±31.3	NS
Total cholesterol (mg/dL)	170.2±56.3	200.5±45.8	0.07
HDL (mg/dL)	43.1±14.6	49.4±15.4	0.17
NLR	3.8±1.9	2.8±1.5	0.10
CRP (mg/dL)	5.1±4.7	1.5±0.7	<0.01
Klotho (pg/mL)	534.8 (435.2-736.5)	905.1 (553.0-1733)	<0.05
TNFα (pg/mL)	1.10 (0.85-1.45)	1.48 (0.88-2.0)	NS
IL10 (pg/mL)	4.16 (0.70-8.06)	10.89 (4.42-25.47)	<0.05
TNFα/IL10	0.29 (0.13-1.69)	0.11 (0.06-0.46)	<0.05

BMI, body mass index; HT, hypertension; DM, diabetes mellitus; PAD, peripheral artery disease; TIA, transient ischemic attack; AAA, abdominal aortic aneurysm; ACEI/ARA2, angiotensin converting enzyme inhibitor/angiotensin receptor antagonist 2; CCB, calcium channels blockers; eGFR, estimated glomerular filtration rate; HDL, high-density lipoprotein; NLR, neutrophil-to-lymphocyte ratio; CRP, c-reactive protein; TNFα, tumor necrosis factor- α; IL10, interleukin 10; NS, not significant.
